# Decreased Proliferation Kinetics of Mouse Myoblasts Overexpressing *FRG1*

**DOI:** 10.1371/journal.pone.0019780

**Published:** 2011-05-16

**Authors:** Steven C. Chen, Ellie Frett, Joseph Marx, Darko Bosnakovski, Xylena Reed, Michael Kyba, Brian K. Kennedy

**Affiliations:** 1 Department of Biochemistry, University of Washington, Seattle, Washington, United States of America; 2 Lillehei Heart Institute and Department of Pediatrics, University of Minnesota, Minneapolis, Minnesota, United States of America; 3 Buck Institute for Age Research, Novato, California, United States of America; Brigham and Women's Hospital, Harvard Medical School, United States of America

## Abstract

Although recent publications have linked the molecular events driving facioscapulohumeral muscular dystrophy (FSHD) to expression of the double homeobox transcription factor *DUX4*, overexpression of *FRG1* has been proposed as one alternative causal agent as mice overexpressing *FRG1* present with muscular dystrophy. Here, we characterize proliferative defects in two independent myoblast lines overexpressing *FRG1*. Myoblasts isolated from thigh muscle of *FRG1* transgenic mice, an affected dystrophic muscle, exhibit delayed proliferation as measured by decreased clone size, whereas myoblasts isolated from the unaffected diaphragm muscle proliferated normally. To confirm the observation that overexpression of *FRG1* could impair myoblast proliferation, we examined C2C12 myoblasts with inducible overexpression of *FRG1*, finding increased doubling time and G1-phase cells in mass culture after induction of *FRG1* and decreased levels of pRb phosphorylation. We propose that depressed myoblast proliferation may contribute to the pathology of mice overexpressing *FRG1* and may play a part in FSHD.

## Introduction

Facioscapulohumeral muscular dystrophy, or FSHD, primarily affects muscles of the face, shoulders and upper arms. It is the third most common muscular dystrophy, following Duchenne muscular dystrophy and myotonic muscular dystrophy, affecting 1 in 20,000 individuals [1]. Onset of muscle weakness in FSHD patients most commonly occurs between puberty and the second decade of life, ultimately leading to patients becoming wheelchair-bound [2], [3], [4]. Compared to the majority of muscular dystrophies, FSHD is unique in its very low rate of any respiratory or cardiac muscle involvement, which is often the eventual cause of death for patients with other forms of muscular dystrophy [5]. As such, patients with FSHD typically live a normal lifespan, but suffer a severely decreased quality of life.

The molecular basis of FSHD is still under debate, although the genetic event linked with FSHD has been identified to be in the subtelomeric region on the long arm of chromosome 4 [6], [7]. This region, denoted as 4q35, contains a series of 3.3 kb tandem repeat elements, which have been termed D4Z4 repeats [8]. Unaffected individuals have 11 to 150 D4Z4 repeats, but patients with FSHD have had this region truncated to 10 or less [9]. Efforts to identify the molecular basis of this disease have been hampered, however, because the truncation associated with FSHD is not within a well-characterized gene coding or promoter region.

Multiple models have been proposed to explain how a D4Z4 repeat truncation is linked to FSHD, reviewed in [10]. The primary model is that the loss of D4Z4 repeats increases expression of a double homeobox transcription factor *DUX4c*, a putative gene centromeric to the D4Z4 repeats and highly homologous to *DUX4* [11], [12], [13]. *DUX4c* has been shown to be up-regulated in FSHD biopsies and primary myoblasts, possibly leading to induction of the *MYF5* myogenic regulator, which serves to inhibit differentiation and activate proliferation [14], [15]. In addition, overexpression of *DUX4* in other cell lines has been shown to cause apoptosis and impair myogenesis in both cell culture models and zebrafish development [16], [17], [18]. A recent chromosomal analysis of affected and unaffected 4q35 alleles has determined that FSHD is linked to a single nucleotide polymorphism located distal to the last D4Z4 repeat [19], which stabilizes the *DUX4* transcript through polyadenylation and may result in elevated protein levels and cytotoxicity via still unknown mechanisms.

A second model proposes that the loss of D4Z4 repeats may increase the available pool of a repressive complex comprised of YY1, HMG2B and nucleolin that is normally bound to D4Z4 repeats. YY1 interacts with Ezh2, a histone lysine methyltransferase, playing a key role in expression of muscle genes during embryonic development [20], [21] and MeCP2, a methyl CpG binding protein involved in Rett syndrome [22]. In addition, YY1 may also be able to interact with the chromatin insulator CTCF [23]. HMGB2 may affect the maintenance of heterochromatic regions by interacting with SP100B and subsequently HP1, establishing higher-order chromatin structures [24], [25]. In contrast, nucleolin may have an opposite effect on heterochromatin formation as it serves to decondense chromatin through displacement of histone H1 [26]. Perturbations in any of these proteins due to loss of D4Z4 repeats resulting in increased chromatin accessibility may cause gene deregulation *in trans* and play a role in the pathogenesis of FSHD.

A third model suggests that D4Z4 may serve as nucleating sites for local transcriptional repression involving the previously mentioned YY1 complex. Loss of D4Z4 may lift repression *in cis* of the 4q35 region and thus the nearby genes *FRG1*, *FRG2* and *ANT1* [27], [28]. Additionally, the identification of a nuclear matrix attachment site (S/MAR) and its disassociation from the nuclear matrix in FSHD patients may change the arrangement of DNA loop domains, causing increased transcription of *FRG1* and *FRG2* [29], [30], [31]. Presently, it is unclear as to which or how many of these many non-exclusive mechanisms play a causal role in the pathogenesis of FSHD.

Previously, it has been observed that there may be increased transcriptional activation of *FRG1*, *FRG2* and *ANT1*, the three genes upstream of the D4Z4 tandem repeat elements, in muscle of human FSHD patients [28]; however, these results were not observed in another patient study [32]. Unfortunately, generating a relevant mouse model to study FSHD has been exceptionally difficult because mice do not have D4Z4 repeats in an analogous chromosomal setting. Acting under the assumption that overexpression of *FRG1*, *FRG2* or *ANT1* plays a causative role in the development of FSHD, transgenic mice were generated expressing each of these individual genes under the human skeletal actin promoter, specific for expression in muscle, which resulted in the identification of a potential mouse model for FSHD. In contrast to transgenic mice overexpressing *FRG2* or *ANT1*, only transgenic mice overexpressing *FRG1* appear to have symptoms characteristic of muscular dystrophy [33]. It should be noted, however, that the *FRG1* transgenic mouse model that resulted in dystrophic phenotypes had FRG1 skeletal muscle protein levels considerably higher than that observed in FSHD patients.

Facioscapulohumeral muscular dystrophy region gene-1 (*FRG1*) is an actin-bundling protein associated with muscle-attachment sites, specifically located to the Z-disc in mature muscle tissue [34], [35]. *FRG1* has been shown to play a crucial and specific role in muscle development of *Xenopus laevis*, further implicating its importance in muscle development and maintenance [36], [37]. Recently, Bodega et al. showed that the *FRG1* gene was prematurely expressed during FSHD myoblast differentiation, thereby suggesting that the number of D4Z4 repeats in the array may affect the correct timing of *FRG1* expression [27].

Based on this work, we hypothesized that the dystrophic phenotype in *FRG1* transgenic mice is caused, at least in part, by decreased proliferation in the muscle satellite cell population, which are the cells responsible for maintaining proper muscle regenerative potential. Satellite cells are often thought of as muscle stem cells, proliferating when there is a need for either muscle repair or growth and then differentiating into skeletal muscle. We have observed that increased levels of FRG1 impair normal satellite cell proliferation and may contribute to disease progression by limiting the pool of cells to repair damaged muscle and/or delaying the kinetics of repair.

## Results

### Expression of FRG1 in skeletal muscle of mice causes dystrophic characteristics

Mice overexpressing *FRG1* have been previously described [33]. In that study, three lines of transgenic mice overexpressing *FRG1* specifically in muscle were generated by using the human skeletal actin promoter to drive transcription of the human *FRG1* cDNA. These mice develop spinal kyphosis and characteristics of muscular dystrophy, including increased fibrosis in muscles, lower body weight, lower muscle weight and decreased cross-sectional area, reduced tolerance to exercise and mis-splicing of specific transcripts associated with myotonic dystrophy. Mice used in the Gabellini et al. study [33] were kindly provided by Dr. Rossella Tupler. After establishing an independent colony with continual back-crossing to C57BL/6 mice, we performed a limited characterization of the *in vivo* phenotypes in their highest *FRG1* expressing line (H-*FRG1* ^TG^) as a confirmation of their reported findings. As expected, these mice begin to show mild spinal kyphosis and reduced body weight by 4 weeks of age, both of which become progressively more severe over time.

**Figure 1:**
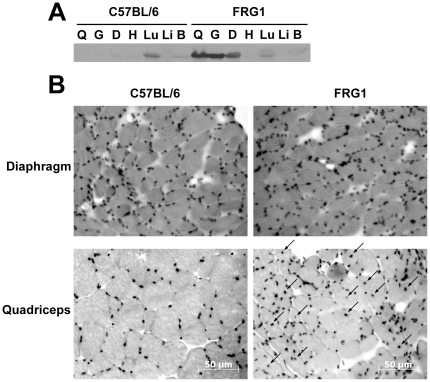
FRG1 expression and dystrophic phenotype of mice. A) Muscles and tissue from either H-*FRG1* ^TG^ transgenic mice (FRG1) or wild-type littermate control (C57BL/6) were collected from 10-week old mice. 300 µg of total protein from tissue lysates isolated from quadriceps muscle (Q), gastrocnemius muscle (G), diaphragm muscle (D), whole heart (H), lung tissue (Lu), liver tissue (Li) and brain tissue (B) were probed with α-FRG1 antibody after SDS-PAGE. B) Diaphragm and quadriceps muscle from 13-week old H-*FRG1* ^TG^ mouse (FRG1) or wild-type littermate control (C57BL/6) stained with hemotoxylin/eosin and viewed under 100× magnification. Arrows note location of centrally located nuclei present in FRG1 cross-section. Scale bar notes 50 µm.

To verify that *FRG1* expression was increased specifically in skeletal muscle, we performed Western blot analysis on lysates generated from a variety of tissues from 10-week old mice using a specific α-FRG1 antibody. Equivalent amounts of total protein were loaded and increased FRG1 protein was detected in all tested skeletal muscles of H-*FRG1* ^TG^ mice compared to wild-type littermate controls (**Figure 1A**). We observed a specific increase in the expression of *FRG1* in skeletal muscles such as quadriceps, gastrocnemius and diaphragm muscle, and no increase of *FRG1* in cardiac muscle or other tissues, confirming the skeletal muscle-specific expression of *FRG1*. Endogenous expression of *FRG1* was detected in the lung, but there was no significant difference in expression between H-*FRG1* ^TG^ mice and wild-type littermate controls.

Muscle weights of collected tissues were measured at different ages and normalized to body weight, to account for runted phenotype of H-*FRG1* ^TG^ mice. Quadriceps and gastrocnemius wet muscle weight comprised a smaller percentage of total body weight in H-*FRG1* ^TG^ mice compared to wild-type mice, but diaphragm weight showed no significant change (data not shown). This observation agrees with previous observations demonstrating that despite *FRG1* expression in all skeletal muscle tissue, only specific muscles may exhibit a dystrophic phenotype [33], [38].

To examine the histology of specific muscles, we collected and cryosectioned muscle tissue from 13-week old H-*FRG1* ^TG^ mice and wild-type littermate controls. Hemotoxylin and eosin staining of these sections showed an increased incidence of centrally located nuclei and increased fiber size variability in affected muscle tissues, namely the quadriceps, but this was not noted in internal muscles such as the diaphragm (**Figure 1B**). From these experiments we find that overexpression of *FRG1* only causes a dystrophic phenotype in a subset of skeletal muscles. We also noted that H-*FRG1* ^TG^ mice were able to survive past 1 year of age (data not shown) despite the worsening of their muscular dystrophy and other associated phenotypes. The apparent sparing of the diaphragm muscle of any measurable defect may possibly replicate the muscle specificity of FSHD in human patients, which similarly does not appear to affect internal muscles. All of these observations confirm the muscular dystrophy phenotype in H-*FRG1* ^TG^ mice as originally characterized.

### Effects of FRG1 expression in mouse-derived myoblasts

In principle, dystrophic phenotypes in muscle can arise from enhanced degeneration, defective regeneration, or both. For instance, myoblasts from mice lacking A-type lamins that exhibit signs of muscular dystrophy plate with high viability and proliferate normally, but ultimately have impaired differentiation [39]. Thus, muscular dystrophies associated with mutations in the A-type lamin gene, *LMNA*, may be associated with decreased satellite cell differentiation, causing depressed regeneration in addition to reduced myofiber stability. As a test for potential regeneration defects in satellite cells of H-*FRG1* ^TG^ mice, we sought to determine whether myoblasts from these mice had altered proliferation or differentiation potential in cell culture. Myoblast cultures were isolated and cultured by standard techniques [40], which, if treated appropriately, are comprised predominantly of proliferating myoblasts and can provide an accurate readout of the regenerative potential of muscle from the H-*FRG1* ^TG^ mice. We speculated that myoblasts from H-*FRG1* ^TG^ mice may have similar defects to those from *Lmna^−/−^* mice.

We isolated and cultured the satellite cell population from affected quadriceps and unaffected diaphragm muscle of 18-week old H-*FRG1* ^TG^ mice and wild-type littermate controls. Since the quadriceps muscle, but not the diaphragm, exhibited characteristics of muscular dystrophy, we speculated that there is a specific defect in satellite cells derived from the thigh. We thus performed a clonal assay on primary isolated myoblasts in order to measure the proliferation rates of the isolated satellite cells.

We quantified transcript levels of *FRG1* in our isolated satellite cells by qPCR, and observed increased levels of *FRG1* transcript in both proliferating and differentiated cultures from H-*FRG1* ^TG^ mice (**Figure S1A**). This was somewhat surprising as the promoter driving *FRG1* expression, the human skeletal actin promoter, is reported to be active only after differentiation. We also performed Western analysis on cell lysates generated from the same satellite cells to observe protein levels of FRG1. Levels of FRG1 protein are increased in both proliferating and differentiated cultures of satellite cells from of H-*FRG1* ^TG^ mice (**Figure S1B**). In addition, FRG1 is detected in satellite cell cultures derived from either diaphragm or thigh muscle (**Figure S1C**).

**Figure 2:**
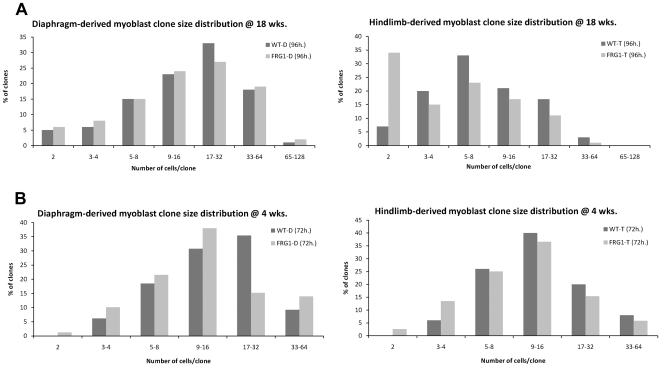
Clonal analysis of mouse-derived myoblasts. A) Myoblasts isolated from diaphragm (D) or thigh (T) of 18-week old H-*FRG1* ^TG^ mice (FRG1) or wild-type littermate controls (WT) were cultured and plated at low density. Cells were fixed at regular time intervals and stained for myosin heavy chain. Total number of nuclei per clone was counted and a representative graph of data from 96-hours post-plating is shown (n = 100). B) Myoblasts isolated from diaphragm (D) or thigh (T) of 4-week old H-*FRG1* ^TG^ mice (FRG1) or wild-type littermate controls (WT) were scored for proliferation as above at 72-hours post-plating (50<n<80).

For clonal analysis, the tissue-derived satellite cells were plated at a very low density, 1000 cells per 10 cm dish, and allowed to grow for a predefined amount of time. At regular intervals, plates were fixed and nuclei were visualized by staining with methylene blue. We also stained for myosin heavy chain (MyHC) as a control to verify that cells did not prematurely differentiate over the course of the clonal assay, in which bFGF and high serum levels were maintained. After fixation and staining, the total number of cells per clone was determined and binned for comparison in a histogram format. Thigh-derived satellite cells from an 18-week old H-*FRG1* ^TG^ mouse show a marked decrease in average clone size compared to those derived from a wild-type littermate control (**Figure 2A**). A significant fraction of these cells show arrest in a 2-cell clone size skewing the distribution compared to the wild-type thigh-derived satellite cells. This effect may be even more dramatic considering that single cell clones were not scored in this assay, as we consider single cell clones may potentially be new clones arising from detached satellite cells floating away from the original clone during mitosis. We replicated these observations with an independent satellite cell culture isolated from 20-week old mouse limbs obtained directly from Dr. Rossella Tupler which were comparable to our 18-week old thigh-derived satellite cells at a similar time point (**Figure S2**). The proliferative defect was not replicated in the diaphragm-derived satellite cells, which show a very similar clone size distribution between the H-*FRG1* ^TG^ and wild-type C57BL/6 littermates. These findings indicate that *FRG1* overexpression leads to a muscle-type specific defect in proliferation, and correlates with the dystrophic phenotype.

To determine whether there is an age-dependent increase in severity of the observed proliferation defect, we isolated and performed a clonal assay on both thigh- and diaphragm-derived satellite cells isolated from 4-week old mice, which appear asymptomatic, to compare to the aforementioned data from more severely symptomatic 18-week old mice. For each of these populations, we scored multiple time points of a clonal assay, to more thoroughly assay the proliferative defect. The clone size distributions of myoblasts from asymptomatic 4-week old mice did not show any significant proliferative defect when compared to their 18-week old counterparts at a similar clone size (**Figure 2B**). To more easily compare the data from these clone size distributions, we obtained the average clone size for each of these populations and calculated the average doubling time of each of these lines. The data show that the proliferative defects associated with *FRG1* overexpression appear to be age-specific, as myoblasts obtained from quadriceps muscle of 4-week old H-*FRG1* ^TG^ mice are indistinguishable from those derived from littermate controls, whereas satellite cells from 18-week old mice H-*FRG1* ^TG^ mice have a severe defect (**Table 1**). These effects are further reflected in the general health of the mice and severity of muscle dystrophy at later ages. It is interesting to observe that by 52-weeks of age, we were unable to successfully culture satellite cells from the thigh muscle of H-*FRG1^TG^* mice, possibly due to either extremely depressed proliferation rates or exhaustion of the satellite cell population. It should also be noted that we never observed any significant differentiation defect in any of our satellite cell cultures (data not shown). We conclude that the overexpression of *FRG1* in muscle tissue causes muscle-type-specific and age-dependent impairment in the ability of satellite cells to proliferate when isolated in cell culture.

**Table 1:** Doubling times derived from clonal assays.

	4 wk. old		18 wk. old		
Hours post-plating	48 h.	72 h.	48 h.	96 h.	144 h.
BL/6-D	18	17.4	19.9	22.2	25.6
FRG1-D	17.4	17.7	25.7	21.9	24.4
BL/6-T	18.6	18.8	23	28.1	26
FRG1-T	18.5	19.4	**31.1**	**33.3**	**28.2**

Average calculated doubling time of myoblasts isolated from 4-week or 18-week old H-*FRG1* ^TG^ mice (FRG1) or wild-type littermate control (BL/6) at varying times post-plating. Doubling times were calculated from average clone size with the following formula: [T *Ln(2)]/Ln(average clone size).

### Inducible FRG1 expression in C2C12 myoblasts

In addition to experiments performed in mouse-derived satellite cell culture, we sought to establish an independent cell culture model system that would be free of any potential artifacts introduced during generation of the H-*FRG1^TG^* mice. Initially, we performed viral transduction of *FRG1* under the CMV promoter in the pMXIH vector as previously described [41]. Findings in early passages after selection indicated a proliferative defect associated with *FRG1* overexpression. Unfortunately, we observed a loss of the proliferative defect over time as measured by BrdU-positive cells (**Figure S3**). The loss of defects in proliferation was accompanied by an increase in cells staining negative for FRG1, suggesting either that expression was being actively silenced or, more likely, high *FRG1* expressing cells were being outcompeted over time by lower expressing cells with a faster rate of proliferation. Given this complication, we adopted another strategy to drive *FRG1* overexpression.

**Figure 3:**
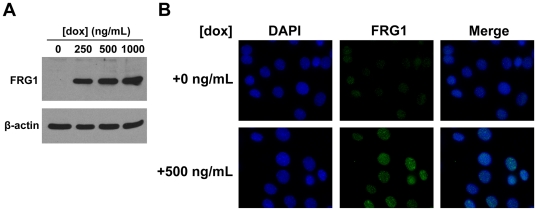
Expression of FRG1 in iC2C12-FRG1 myoblasts. A) Western blot of lysates from iC2C12-FRG1 myoblasts before induction with doxycycline or after induction for 24 hours with concentrations ranging from 250 ng/mL to 1000 ng/mL using α-FLAG antibody. β-actin loading control shown below. B) Localization of FRG1 by immunofluorescence in iC2C12-FRG1either uninduced or induced with 500 ng/mL doxycycline for 24 hours. DAPI stain is represented in the blue channel and α-FRG1 antibody staining is represented in the green channel.

We chose to employ an inducible system to control expression of *FRG1* that would allow us to turn on expression when needed. We utilized C2C12 myoblasts that have an integrated cassette expressing FLAG-tagged *FRG1* under a tetracycline-responsive promoter (iC2C12-FRG1) generated as previously described and kindly provided to us by Dr. Michael Kyba [17]. *FRG1* expression is induced in iC2C12-*FRG1* myoblasts by the addition of doxycycline and levels of induction can be modulated by adjusting levels of the drug. There is no detectable expression of *FRG1* in uninduced cells as assayed by Western blot of whole cell lysates using an α-FLAG antibody. In comparison, robust expression of *FRG1* was observed under a variety of induction conditions ranging from 250 to 1000 ng/mL doxycycline (**Figure 3A**).

FRG1 is believed to play a role in post-transcriptional mRNA processing and localizes to nuclear Cajal bodies [42], therefore we performed immunofluorescence to investigate whether FRG1 was properly localized in iC2C12-FRG1 myoblasts after induction. iC2C12-FRG1 myoblasts were cultured on glass coverslips and cells were grown for 24 hours in the presence or absence of doxycycline followed by fixation and staining. Staining with DAPI and an α-FLAG antibody revealed nuclear localization of FRG1-FLAG following induction (**Figure 3B**). There was some variation in magnitude of expression of FRG1-FLAG on a cell-to-cell basis, but we observed robust partially punctuate staining in the nucleus, consistent with possible localization to Cajal bodies.

Interestingly, we observed in our initial characterization of induced iC2C12-*FRG1* myoblasts a similar phenomenon as the virally transduced C2C12 cells in that after numerous passages in the presence of doxycycline, induced cells showed much lower expression levels of FRG1-FLAG (**Figure S4**) and likely a loss of any proliferative defect. A similar explanation is likely that lower *FRG1* expressing cells in this non-clonal population ultimately outcompete higher expressing cells and take over the culture.

### Characterizing defects in proliferation following FRG1 expression in iC2C12-FRG1 myoblasts

**Figure 4:**
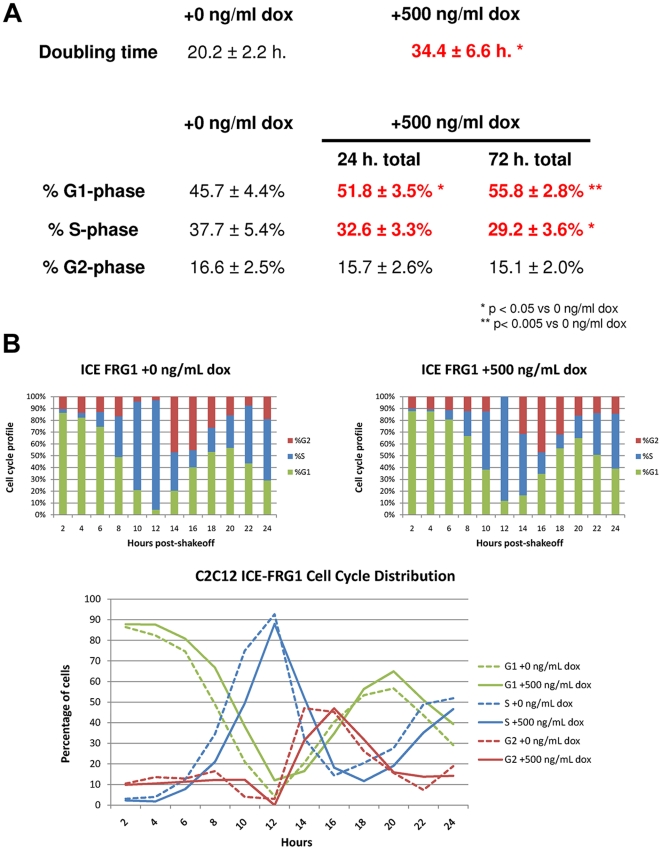
Characterization of proliferation defect in iC2C12-FRG1 myoblasts. A) Mass culture doubling times calculated from hemocytometer counts of iC2C12-FRG1 myoblasts with or without induction of expression by doxycycline over 120 hours. Below are the cell cycle profiles of uninduced iC2C12-FRG1 myoblasts as well as after 24 hours and 72 hours in the presence of doxycycline. Cell cycle profiles were calculated from DNA content analysis by flow cytometry on proliferating myoblasts that were fixed and DAPI stained. *denotes statistical significance p<0.05, **p<0.005. B) Cell cycle profiles of synchronized iC2C12-*FRG1* myoblasts over 24 hours in the absence or presence of doxycycline showing fraction of cells in G1, S or G2-phase. Comparison of data is plotted in a line graph fashion showing G1 in green, S in blue and G2 in red to demonstrate the generalized lag exhibited by iC2C12-*FRG1* myoblasts expressing *FRG1*. Uninduced iC2C12-FRG1 myoblasts are graphed with a dashed line, while iC2C12-*FRG1* myoblasts with doxycycline-induced *FRG1* expression are graphed with a solid line. n>20,000 cells analyzed for each time point.

To investigate the proliferation defect caused by *FRG1* overexpression, we conducted a number of assays in iC2C12-FRG1 myoblasts. We first assayed the proliferation rates of cells in mass culture in the presence or absence of doxycycline. In order to determine growth rates, identical numbers of cells were plated in wells of a 24-well plate and at regular time intervals the total number of cells per well were counted using a hemocytometer. We determined that induction of FRG1-FLAG in iC2C12-FRG1 myoblasts has a negative effect on their proliferation rate, as shown by the increased doubling time when grown in doxycycline (**Figure 4A**). Naïve C2C12 myoblasts were not affected by exposure to doxycycline (data not shown), demonstrating that the phenotype is likely specific to *FRG1* overexpression.

To further investigate the proliferative defect of iC2C12-FRG1 myoblasts expressing *FRG1*, we utilized flow cytometry to determine the fraction of cycling cells in specific phases of the cell cycle. Actively proliferating iC2C12-FRG1 myoblasts grown in either the presence or absence of doxycycline were fixed and stained with DAPI. Subsequent flow cytometry analysis of the cells for DNA content indicated an increased fraction of FRG1 overexpressing iC2C12-FRG1 myoblasts in G1-phase, coupled with a corresponding decrease in the fraction of cells in S-phase (**Figure 4A**). These findings suggest that cells overexpressing FRG1 are delayed in transit through G1. We believe that this shift in cell cycle profile may at least in part explain the gross proliferation defect that we have observed.

One potential weakness of just looking at a cell cycle phase distribution is that absolute cell cycle length is indeterminate in a mass culture cell cycle profile. To address this issue, we performed a mitotic shakeoff assay to synchronize proliferating iC2C12-FRG1 myoblasts so that transit through specific cell cycle phases could be measured. Since cells progressing through mitosis are less adherent to culture dishes, this technique provides a way to synchronize cells in the absence of any drugs to mediate cell cycle arrest. After being plated in the presence or absence of doxycycline for a period of 24 hours, mitotic cells were isolated by mild shaking and rocking for a period of 20 minutes to generate synchronized cultures. Fixation at 2-hour intervals followed by subsequent analysis by flow cytometry for DNA content was performed to track cells as they progressed through the cell cycle. We observed that FRG1 overexpressing cells exhibited a consistent 1–2 hour delay in exit from G1 and entry into S-phase (**Figure 4B** **&** **Table 2**). This finding is consistent with the FACS analysis of asynchronous cells, which indicated that a higher percentage of FRG1 overexpressing cells were in G1 phase. It should be noted that it is difficult to monitor progression beyond one cell cycle in this assay since the iC2C12-FRG1 myoblasts rapidly lose synchronicity.

**Table 2:** Raw cell cycle profile data.

	2	4	6	8	10	12	14	16	18	20	22	24
**ICE FRG1 −dox**												
*%G1*	86.41	82.4	74.58	48.95	21.03	4.31	20.55	40.35	53.35	56.66	43.65	29.16
*%S*	3.11	4	12.52	34.53	74.94	92.66	32.41	14.43	20.47	27.68	48.95	51.91
*%G2*	10.48	13.6	12.9	16.52	4.03	3.03	47.04	45.22	26.18	15.66	7.4	18.93
**ICE FRG1 +dox**												
*%G1*	87.83	87.67	80.81	66.74	38.25	12.04	16.5	34.75	56.41	64.96	50.97	39.37
*%S*	2.31	1.82	7.83	21.04	49.45	87.96	52.06	18.24	11.67	19.11	35.2	46.6
*%G2*	9.86	10.51	11.36	12.22	12.3	0	31.44	47.01	31.92	15.93	13.83	14.23

Values from WinCycle analysis of synchronized ICE-FRG1 myoblasts in the presence or absence of doxycycline-induced FRG1 expression showing fraction of cells in G1, S or G2-phase of the cell cycle plotted in table form to emphasize differences. N>20,000 analyzed for each timepoint.

### Cell cycle specific factors are affected by FRG1 expression

**Figure 5:**
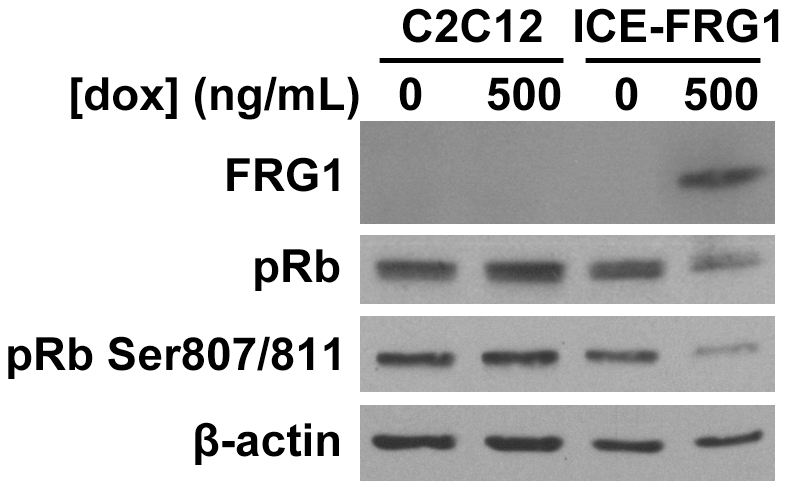
Phosphorylation of pRb is perturbed by FRG1 expression. Western blot analysis for pRb, pRb Ser807/811 and FRG1 in iC2C12-FRG1 cells grown in the presence or absence of doxycycline. β-actin loading control shown below.

The retinoblastoma protein (pRb) is one of the primary regulators of the mammalian cell cycle, inhibiting E2F-dependent transcription and maintaining cells in G1-phase when hypophosphorylated. Phosphorylation by cyclin-dependent kinases interferes with the capacity of pRb to repress E2F-dependent transcription, permitting S-phase entry [43], [44]. Since *FRG1* overexpression led to increased G1 phase occupancy, we examined the phosphorylation state of pRb as an indicator of these altered cell cycle kinetics. We observe via Western blot that compared to naïve C1C12 or uninduced iC2C12-FRG1 myoblasts, levels of 807/811 phosphorylated pRb are lower in induced iC2C12-FRG1 myoblasts (**Figure 5A**). Total levels of pRb appear lower, however this is likely an artifact of the antibody used, since it is known to have somewhat greater affinity for hyperphosphorylated pRb. Decreased pRb phosphorylation is consistent with a decreased proliferation rate and increased G1 phase occupancy.

## Discussion

In this study, we demonstrate decreased proliferation rates of myoblasts expressing *FRG1*, an attribute that could contribute to the long term reduction in muscle regenerative potential and muscular dystrophy observed in transgenic mice overexpressing *FRG1*. We have verified expression and muscular dystrophy in the H-*FRG1* ^TG^ mouse and seen that thigh-derived myoblasts, but not diaphragm-derived myoblasts, from these animals demonstrate a proliferative defect by clonal analysis. We also find that induction of *FRG1* in myoblasts by a tetracycline-responsive system negatively affects proliferation as determined in cell cycle profiles measured by flow cytometry and hypophosphorylation of pRb.

Reduced myoblast proliferation is not commonly linked to muscular dystrophy, which is more classically attributed to death of muscle fibers, such as in Duchenne muscular dystrophy [45], or a combination of enhanced fiber degeneration and defective differentiation kinetics such as in *Lmna* ^−/−^ mouse models [39], [46]. However, there have been reports of depressed proliferation kinetics in Duchenne muscular dystrophy myoblasts, compounding the existing mechanisms of muscular dystrophy [47]. Since the proliferation defect gets more severe in myoblasts isolated from H-*FRG1* ^TG^ mice of increasing age, it is difficult to differentiate between two models: (1) that the proliferation defect precedes onset of the dystrophic phenotype or (2) that the defect derives from reduced satellite potential with age resulting from increasing strain on satellite cells to repair damage.

Our findings in C2C12 cells may indicate that a cell cycle defect can occur as a primary result of *FRG1* overexpression. However, it ultimately remains unclear precisely how FRG1 impacts cell cycle progression. FRG1 has been shown to localize to Cajal bodies in the nucleus, where it is reported to regulate RNA processing [42], [48]. Misprocessing of RNA transcripts has also been linked to myotonic dystrophy [49], [50]. One possible hypothesis for induction of G1 arrest by FRG1 involves altered splicing of transcripts encoding cell cycle components. For instance, altered processing of the cyclin E RNA produced different isoforms of the protein with different affinities for Cdk2 [51], [52], [53].

One contentious observation regarding overexpression of *FRG1* in patients with FSHD is that other research groups have been unable to replicate the results published showing increased *FRG1* transcript in affected muscle [28]. Some studies of myoblasts subjected to microarray analysis or measuring RNA transcription of *FRG1* have not yielded any results showing an increase of *FRG1* transcript [54], [55], while similar experiments with qRT-PCR done in other groups have shown an increased trend [27]. Recently, however, Bodega et al. reported that overexpression of *FRG1* in muscle biopsies is not a uniform finding and may depend on the composition and age of the muscle biopsy, as *FRG1* is only upregulated during an early state of differentiation into myotubes. Regardless of whether the levels of *FRG1* are increased or not in FSHD patients, the H-*FRG1* ^TG^ mouse is a valuable tool for studying the mechanics of muscular dystrophy, though it is important to note that *FRG1* expression in these mice is many fold higher than observed in any human patients. Ultimately, FRG1 remains relatively poorly characterized and our findings may help to further elucidate its function.

It has been reported that myoblasts isolated from human muscle biopsies exhibit a morphological difference upon differentiation [56]. The authors observed that, compared to differentiated wild-type myotubes, FSHD myotubes were thinner, less branched, more disorganized and were comprised of fewer myoblasts as measured by total number of nuclei per myotube. Converse to our findings, they did not note any proliferation defects in their myoblast cultures, which may be attributed to differences in their isolation technique, sample sources and/or assay techniques. Regardless of the caveats, this raises the possibility of a compound defect in both proliferation and differentiation as a mechanism for the development of FSHD.

One disparity in our data may be the difference observed between the proliferative defect described in mass culture (Figure 4A) and that seen by mitotic shakeoff analyzed by flow cytometry (Figure 4B). Although the difference seen by mitotic shakeoff is less severe than that observed by mass culture doubling time, it is worth noting that the cells are kept under different conditions in each of these assays. In mass culture, myoblasts find themselves in much denser conditions with self-conditioned media. In the mitotic shakeoff, myoblasts are in a much less dense environment and exposed to fresh media and growth factors. It is possible that, given enough time, the cell cycle profiles of myoblasts isolated by mitotic shakeoff would exhibit a more severe defect with increased cell density, but it is impossible to determine this within the time frame whereby cells remain synchronized.

In summary, our experiments have demonstrated the expression of *FRG1* in mouse muscle causes a tissue-specific and age-dependent proliferative defect in the satellite cell population, possibly playing a part in the development of muscular dystrophies and FSHD in humans.

## Materials and Methods

### Animal care and genotyping

High-expressing *FRG1* transgenic mice (H-*FRG1* ^TG^) were kindly provided by Dr. R. Tupler [33]. DNA was extracted from tail samples by digestion with TENS solution (50 mM Tris pH 7.5, 100 mM EDTA pH 8.0, 400 mM NaCl, 0.4% SDS, proteinase K 0.5 mg/mL) followed by ethanol precipitation of genomic DNA. PCR genotyping of FRG1 mice was performed with the following primers: HSA-FRG1-5′ 5′-GAT CTA GCG GCC GCC ATG GCC GAG TAC TCC TAT GTG AAG TCT-3′ and HSA-FRG1-3′ 5′-GCG CGC TTA ATT AAT CAC TTG CAG TAT CTG TCG GCT TTC A-3′. Mice were bred and maintained under specific pathogen-free conditions. All experiments were performed in compliance with the University of Washington Institutional Animal Care and Use Committee protocol #2362-04.

### Analysis of FRG1 expression

Tissue lysates were generated from quadriceps, gastrocnemius, diaphragm, heart, liver, lung and brain tissue isolated from H-*FRG1* ^TG^ mice and wild-type littermate controls. Homogenization was done in 5 mL of ice-cold RIPA buffer supplemented with protease inhibitors (50 mM Tris-HCl pH 7.4, 150 mM NaCl, 1% NP-40, 0.25% deoxycholate, 1 mM Na3VO4, 1 mM NaF, 1 mM PMSF, 1 µg/mL aprotinin, 1 µg/mL leupeptin) per gram of tissue for 30 seconds using the Omni-Tip system (OMNI International). Samples were incubated on ice for 30 minutes, spun down at 16,500 rcf for 15 minutes at 4°C, followed by transferring of supernatant and recentrifugation for an additional 10 minutes to generate tissue lysates.

RNA was isolated from both proliferating and differentiated mouse muscle satellite cell cultures using the RNAqueous kit (Ambion Inc). qPCR was performed using primers as previously described [33]. Cell lysates were also generated from the aforementioned muscle satellite cell cultures by homogenization in RIPA buffer as described above.

### Histological analysis of muscle

Quadriceps, gastrocnemius, soleus and diaphragm were isolated from H-*FRG1* ^TG^ mice and wild-type littermate controls, blotted briefly on filter paper and weighed on an analytical balance. Tissues were mounted in Tissue-Tek OCT compound (Sakura Finetek), frozen in a liquid nitrogen-cooled bath of isopentane and stored at −80°C. Cryosections were collected using a Leica CM1850 for hemotoxylin and eosin staining. Stained sections were viewed and photographed under 100× magnification on a Zeiss Axiovert 200 M. Analysis of images was performed using AxioVision v4.3.

### Isolation of mouse muscle satellite cells and cell culture

To isolate satellite cells, 4-week and 18-week old FRG1 or wild-type mouse thigh or diaphragm muscle tissue was dissected away and placed in chilled Growth Media (GM) consisting of Ham's F10C+15% horse serum (Atlanta Biologicals Lot D0195)+50 µg/ml gentamicin with the addition of 0.25 µg/ml Fungizone. Connective tissue and fat were dissected away from muscle tissue under a dissecting microscope and muscle was weighed on an analytical balance. Isolation of the myoblastic population was performed as previously described [40], with the following modifications and details. Diced muscle was minced for 2 minutes using curved-tip scissors and treated with 50 µL of 0.05% trypsin-EDTA (Gibco) diluted in Hank's saline per mg of tissue. The tissue/trypsin mixture was incubated at 37°C and pipetted thoroughly every 5 minutes for 20 minutes total. The trypsinized tissue mixture was then passed through a 70 µm cell strainer (BD Falcon) to reduce fibroblast contamination and the strainer was rinsed with an additional 100 µL of F10C per mg of tissue. The flowthrough was then centrifuged at 800 rpm (Sorvall Legend T) for 5 minutes and plated on gelatin-coated dishes in GM+6 ng/ml basic fibroblast growth factor (bFGF)+0.25 µg/ml Fungizone [40]. The satellite cell cultures were observed carefully and differentially passaged to eliminate any fibroblast contamination over 5 passages. To differential passage, satellite cells were treated to a mild trypsinization and observed under a microscope until the round satellite cells were seen detaching from the plate. Because of their morphological differences, satellite cells will detach from the dish before the flattened and larger fibroblasts. Once the satellite cells were observed detaching, the trypsin solution was immediately removed and plated to isolate the satellite cell population.

The established mouse-derived satellite cell cultures were cultured on 10 cm collagen-coated tissue culture plates in GM with 4 ng/ml bFGF. Cells were supplemented with fresh bFGF every 12 hours to inhibit differentiation of satellite cells into myocytes. Passaging occurred when cells reached 5×10^5^ cells in a 10 cm dish by rinsing with Saline A (10 mM dextrose, 30 mM HEPES, 3 mM KCl, 130 mM NaCl, 1 mM Na_2_HPO_4_-7H_2_O), trypsinization and replating at 5×10^4^ in a new 10 cm dish.

iC2C12-FRG1 myoblasts were generated as described and generously provided by Dr. Michael Kyba at the University of Texas Southwestern [57]. iC2C12-FRG1 myoblasts were maintained in DMEM (Hyclone)+10% FBS (Atlanta Biologicals Lot A0087) taking care to passage before confluency to avoid loss of the myoblastic population. Induction of FRG1-FLAG in iC2C12-FRG1 myoblasts was achieved by incubating cells with 500 ng/mL doxycycline for 24 hours.

### Clonal analysis of proliferation

To perform clonal assays, proliferating satellite cells were trypsinized and plated at 1000 cells per 10 cm dish in GM with 6 ng/mL bFGF to prevent premature differentiation. Cells were left undisturbed to minimize the formation of satellite clones and fixed with AFA (50% ethanol, 5% formalin, 5% acetic acid) at regular intervals. Myoblast clones were then immunostained for myosin heavy-chain (MyHC) using mouse monoclonal MF-20, biotinylated rabbit anti-mouse IgG, streptavidin and biotinylated horseradish peroxidase (Vector Labs, Inc.), followed by counterstaining with 1% methylene blue as previously described [39]. Clones were then scored by visualization at 25×/50× magnification on a dissecting microscope for both number and percent differentiation. Doubling times were calculated with the formula “Doubling Time = [Time post-plating *Ln(2)]/Ln(average clone size)”.

### Viral transduction of C2C12 myoblasts

Expression vectors for *FRG1* were generated in the pMXIH vector as described previously [41]. 293T cells were transfected with either pMXIH or pMXIH-FRG1 in combination with the ϕ-ampho packaging plasmid using calcium phosphate. Virus-containing media were filtered through a 0.45 µm filter and applied to C2C12 myoblasts to generate C2C12-pMXIH vector control myoblasts and C2C12-FRG1 myoblasts which should stably express FRG1 protein.

### Flow cytometry and other proliferation assays

To assay doubling time of iC2C12-FRG1 myoblasts, proliferating cells were grown in the presence or absence of doxycycline to induce expression of *FRG1*. 2000 cells were plated per well of a 24-well dish. One well of cells plated in this dish per condition was trypsinized every 24 hours over 120 hours and total cell number was counted in triplicate on an improved Neubauer hemocytometer. Cell number was graphed and exponential curve fit was performed using Microsoft Excel to determine the growth constant and calculate the doubling time.

For BrdU staining of virally-transduced C2C12 myoblasts, after viral transduction, cells were plated on glass coverslips and pulsed with BrdU cell proliferation reagent (Amersham) for 60 minutes. Cells were then fixed in 4% formalin, permeabilized with 0.5% Triton X-100 in PBS and blocked in 5% goat serum, 5% horse serum, 0.2% Tween-20, 0.2% fish skin gelatin in PBS. α-BrdU mouse antibody (BD Biosciences) was diluted 1∶350 in PBS and incubated for 1 hour at 37°C followed by goat α-mouse antibody conjugated to AlexaFluor488 diluted 1∶400. Coverslips were mounted using Vectashield (Vector Labs).

For flow cytometry assays, proliferating iC2C12-FRG1 myoblasts were also plated in 10 cm dishes for cell cycle profile analysis. After trypsinization, cell pellets were resuspended in DAPI solution (2 mM CaCl_2_, 22 mM MgCl_2_ 0.1 mg/mL BSA, 0.1% Nonidet P-40, 10 µg/mL DAPI, 10% DMSO) and run on an InFlux flow cytometer (Cytopeia). 20,000 cells were run through the flow cytometer measuring DNA content. Analysis was done using WinCycle (Phoenix Flow Systems) to determine cell cycle phase distributions as previously described [58].

For determining initial effects of FRG1 expression in synchronized cells, proliferating iC2C12-FRG1 myoblasts either exposed to doxycycline for 24 hours previously or grown in the absence of doxycycline were synchronized by mitotic shakeoff as described [59]. Three 10-cm dishes were tapped vigorously against a hard surface to shake off mitotic cells, incubated with gentle rocking for 20 minutes to prevent reattachment, then tapped vigorously again to further dislodge mitotic cells, and finally the medium containing mitotic cells was centrifuged and plated in a single 60-mm dish. Twelve 60-mm dishes of synchronized cells were collected and placed either in the presence or absence of doxycycline. Every 2 hours after the initial plating, cells were trypsinized and pellets were fixed and resuspended in the same DAPI solution used for flow cytometry to cover a total period of 24 hours. DNA content was measure as previously mentioned using flow cytometry, with 20,000 cells being analyzed.

### Immunofluorescence and immunoblotting

iC2C12-FRG1 myoblasts with or without the induction of FRG1-FLAG were grown on round glass coverslips, fixed in a 4% formalin solution and stained with mouse monoclonal α-FLAG antibody (Sigma) at 1∶10,000 dilution. For cell lysates, proliferating satellite cells or iC2C12-FRG1 myoblasts were lysed in RIPA buffer at 4°C, incubated on ice for 30 minutes, and centrifuged at 16,500 rcf for 10 minutes to remove cell debris. Cell lysates were run on a 10% SDS-PAGE gel, transferred to nitrocellulose overnight at 4°C in a Tris-glycine buffer and incubated overnight at 4°C with one of the following primary antibodies. Monoclonal α-FRG1 at 1∶1000 (Abnova clone 4A5), mouse monoclonal pRb at 1∶1000 (BD Pharmingen clone 14001A), rabbit anti-mouse α-pRb Ser795 at 1∶1000 (Cell Signaling), rabbit anti-mouse α-pRb Ser807/811 at 1∶1000 (Cell Signaling), or mouse monoclonal α-β-actin at 1∶10000 (Abcam). Western blotting was completed the next day using the corresponding secondary antibody of donkey anti-rabbit HRP at 1∶10000 (Amersham Biosciences) or rabbit anti-mouse HRP at 1∶10000 (Amersham Biosciences). Blots were visualized using ECL substrate (PerkinElmer) on Kodak Biomax Light (Sigma) film.

## Supporting Information

Figure S1 **Examination of** ***FRG1*** **expression in muscle-derived myoblasts.** A) Levels of *FRG1* in 20-week old diaphragm-derived myoblasts from either H-*FRG1* ^TG^ (FRG1) or wild-type littermate control (C57BL/6) as assayed by qPCR normalized to GAPDH. Proliferating cultures were judged to have less than 5% differentiated cells while differentiated cultures exhibited greater than 70% differentiation. *indicates no expression detected. B) Western analysis on proliferating and differentiated diaphragm-derived myoblast cultures from 20-week old mice as described above probing for total FRG1 levels. β-actin loading control shown below. C) Western analysis on proliferating satellite cell cultures from diaphragm or thigh of 4-week old mice probing for total FRG1 protein levels. β-actin loading control shown below.
(TIF)

Figure S2 **Additional clonal analysis of mouse-derived myoblasts.** Myoblasts isolated from hindlimb of 18-week old H-*FRG1* ^TG^ (FRG1) or wild-type littermate controls (WT) were cultured and plated at low-density. Total number of nuclei per clone were counted at 120-hours post-plating (n = 100). Similarly myoblasts isolated from dissected hindlimbs of 20-week old H-*FRG1* ^TG^ mouse (FRG1) or wild-type littermate controls (WT) were subjected to this procedure in the lower figure showing total number of nuclei per clone at 120-hours post-plating (n = 13 for FRG1 line, n = 73 for WT line).
(TIF)

Figure S3 **Loss of proliferative defect in virus-transduced C2C12 myoblasts.** C2C12 mouse myoblasts transduced with either vector control (pMXIH) or a *FRG1*-expressing construct (*FRG1*) were scored for incorporation of BrdU after 60-minute pulse to determine % of S-phase cells. Transduced myoblasts show proliferative defect at early passages (passage 8) but lose the phenotype over time.
(TIF)

Figure S4 **Loss of FRG1 expression in iC2C12-FRG1 myoblasts over time.** iC2C12-FRG1 myoblasts were cultured and maintained with or without the presence of 500 ng/mL doxycycline to induce *FRG1* expression. Immunofluorescence with an α-FLAG antibody (green) and DAPI staining (blue) reveals loss of *FRG1* expression after ∼20 passages under induction conditions, but robust expression with an acute induction of 24 hours.
(TIF)
