# Supplementary figures and images for: Decreased Proliferation Kinetics of Mouse Myoblasts Overexpressing FRG1
Source: PLoS One. 2011 May 16;6(5):e19780. doi: 10.1371/journal.pone.0019780 (PMC3095625; doi:10.1371/journal.pone.0019780)

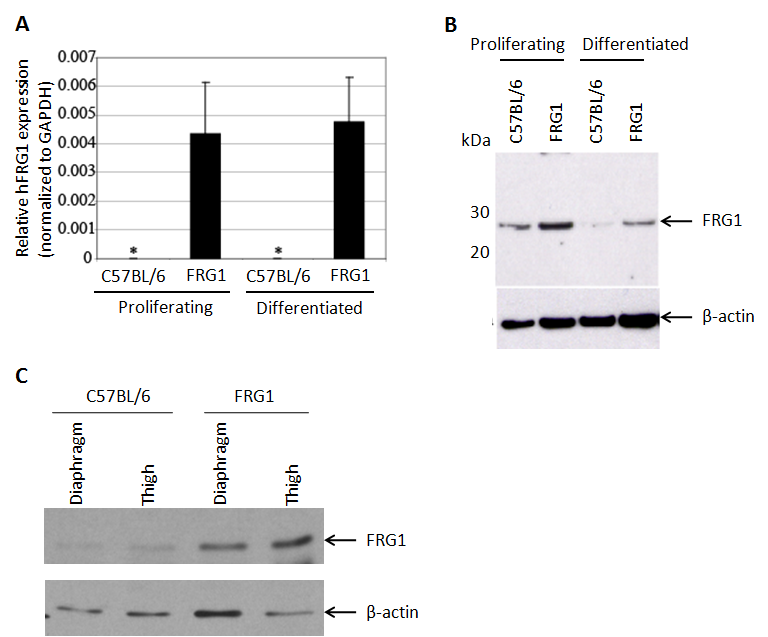

Supplement: Figure S1 — Examination of FRG1 expression in muscle-derived myoblasts. A) Levels of FRG1 in 20-week old diaphragm-derived myoblasts from either H-FRG1 TG (FRG1) or wild-type littermate control (C57BL/6) as assayed by qPCR normalized to GAPDH. Proliferating cultures were judged to have less than 5% differentiated cells while differentiated cultures exhibited greater than 70% differentiation. *indicates no expression detected. B) Western analysis on proliferating and differentiated diaphragm-derived myoblast cultures from 20-week old mice as described above probing for total FRG1 levels. β-actin loading control shown below. C) Western analysis on proliferating satellite cell cultures from diaphragm or thigh of 4-week old mice probing for total FRG1 protein levels. β-actin loading control shown below. (TIF) [file pone.0019780.s001.tif]

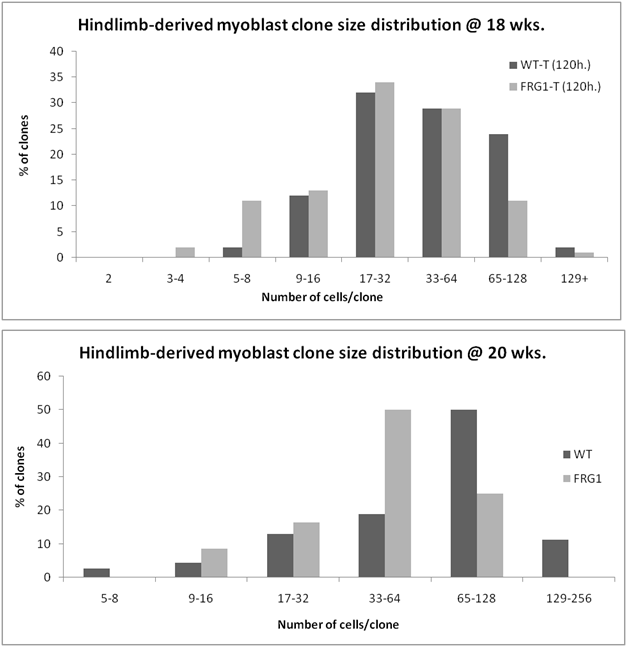

Supplement: Figure S2 — Additional clonal analysis of mouse-derived myoblasts. Myoblasts isolated from hindlimb of 18-week old H-FRG1 TG (FRG1) or wild-type littermate controls (WT) were cultured and plated at low-density. Total number of nuclei per clone were counted at 120-hours post-plating (n = 100). Similarly myoblasts isolated from dissected hindlimbs of 20-week old H-FRG1 TG mouse (FRG1) or wild-type littermate controls (WT) were subjected to this procedure in the lower figure showing total number of nuclei per clone at 120-hours post-plating (n = 13 for FRG1 line, n = 73 for WT line). (TIF) [file pone.0019780.s002.tif]

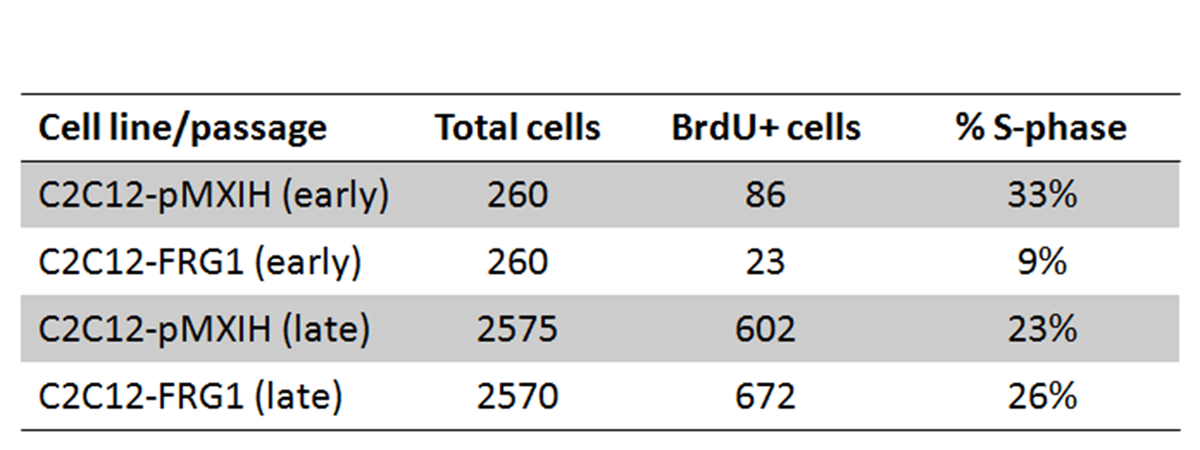

Supplement: Figure S3 — Loss of proliferative defect in virus-transduced C2C12 myoblasts. C2C12 mouse myoblasts transduced with either vector control (pMXIH) or a FRG1-expressing construct (FRG1) were scored for incorporation of BrdU after 60-minute pulse to determine % of S-phase cells. Transduced myoblasts show proliferative defect at early passages (passage 8) but lose the phenotype over time. (TIF) [file pone.0019780.s003.tif]

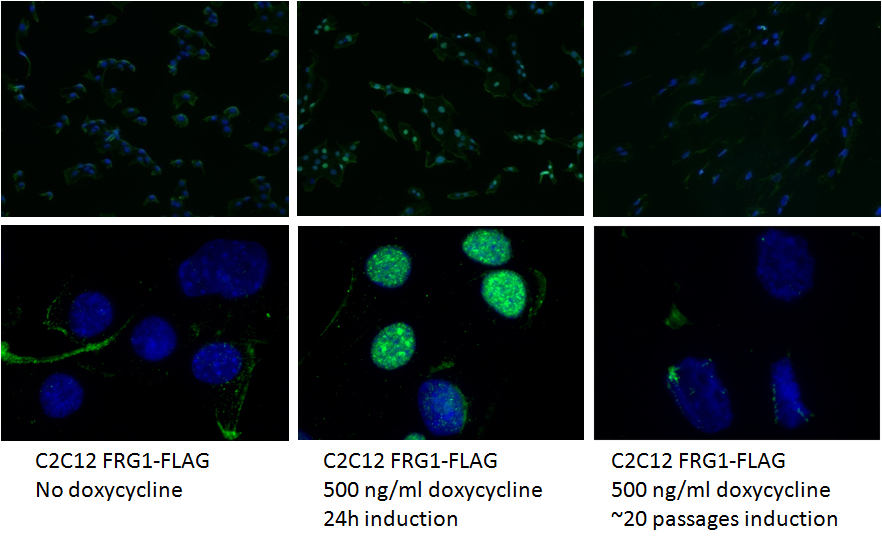

Supplement: Figure S4 — Loss of FRG1 expression in iC2C12-FRG1 myoblasts over time. iC2C12-FRG1 myoblasts were cultured and maintained with or without the presence of 500 ng/mL doxycycline to induce FRG1 expression. Immunofluorescence with an α-FLAG antibody (green) and DAPI staining (blue) reveals loss of FRG1 expression after ∼20 passages under induction conditions, but robust expression with an acute induction of 24 hours. (TIF) [file pone.0019780.s004.tif]
